# Primary Bone Lymphoma: A Case Series and Review of Literature

**DOI:** 10.1155/2020/4254803

**Published:** 2020-04-10

**Authors:** Poorva Bindal, Aakash Desai, Lukas Delasos, Sudhanshu Mulay, James Vredenburgh

**Affiliations:** ^1^Department of Hematology and Oncology, Beth Israel Deaconess Medical Center, Boston, MA, USA; ^2^Department of Internal Medicine, University of Connecticut Health Center, Farmington, CT, USA; ^3^Department of Hematology and Oncology, Smilow Cancer Hospital at St. Francis, Hartford, CT, USA

## Abstract

Primary bone lymphoma (PBL) is a subtype of lymphoma that exclusively affects skeletal tissue. Despite the relatively common involvement of skeletal structures as a manifestation of non-Hodgkin's lymphoma (NHL), primary and exclusive involvement of the skeletal system is rare. The prevalence of PBL is estimated to be 3–7% amongst primary bone tumors and less than 2% amongst all lymphomas in adults. However, the definition of primary bone lymphoma has been inconsistent over time. Within our institution, we identified four cases of primary bone lymphoma based on diagnostic criteria formed from the general consensus of multiple organizations, including the World Health Organization (WHO) and International Extranodal Lymphoma Study Group (IELSG). Here, we discuss the distinct characteristics amongst these cases in addition to performing a systematic review of current literature regarding this lymphoproliferative entity.

## 1. Introduction

Primary bone lymphoma (PBL) is a subtype of lymphoma that exclusively affects skeletal tissue. Despite the relatively common involvement of skeletal structures as a manifestation of non-Hodgkin's lymphoma (NHL), primary and exclusive involvement of the skeletal system is rare [1]. The prevalence of PBL is estimated to be 3–7% amongst primary bone tumors and less than 2% amongst all lymphomas in adults [2].

The definition of primary bone lymphoma has been inconsistent over time. The previous World Health Organization (WHO) classification of soft tissues and bone tumors defined PBL as either single osseous lesions without regional lymph node involvement, or tumor involvement of multiple osseous sites without associated visceral or lymph node disease [3]. However, there is no clearly defined criteria for this malignancy outlined in newer WHO classifications. In the retrospective study conducted by the International Extranodal Lymphoma Study group (the IELSG 14 study), authors defined that only cases with a clear bone origin are classified as primary bone lymphomas, i.e., either a single bony lesion, with or without involvement of regional lymph nodes or multiple bony lesions without nodal or visceral disease, defined as “multifocal osseous lymphoma” or “polyostotic lymphoma”. This definition corroborates with the prior WHO classification; thus, the general consensus amongst clinicians seems to be in line with these criteria.

Here, we describe a case series of four patients from our institution diagnosed with primary bone lymphoma based on the aforementioned diagnostic criteria, in addition to discussing distinct characteristics amongst these cases.

### 1.1. Case Series

#### 1.1.1. Case 1

A 60-year-old female presented to the clinic with complaints of progressive swelling and dull aching pain over the mid-tibia in her left lower extremity. Of note, she had a past medical history of bilateral breast carcinoma in 2003 (Stage I, Grade III invasive ductal carcinoma of the left breast and carcinoma in situ of the right breast) that was estrogen receptor (ER) positive, progesterone receptor (PR) positive, and HER2 negative with absence of BRCA mutation treated with bilateral mastectomies and hormonal therapy.

**Figure 1:**
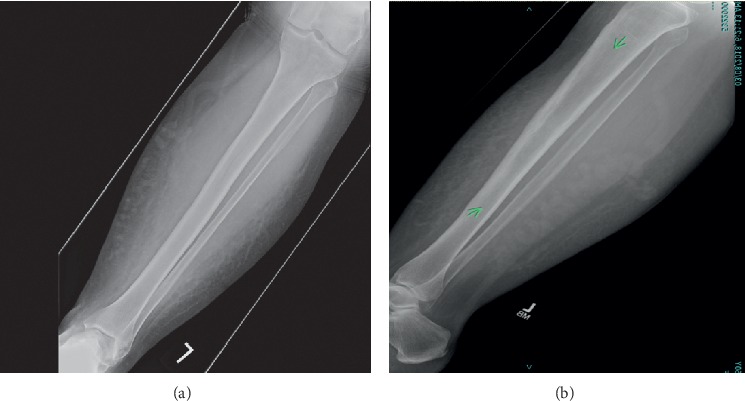
X-ray of the tibia fibula. (a) Antero-posterior (AP) view. (b) lateral view.

**Figure 2:**
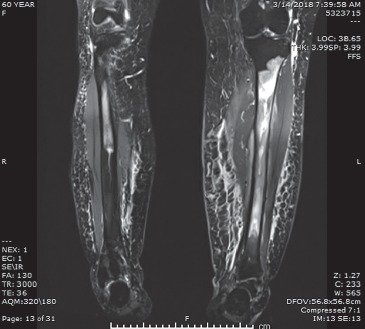
MRI of left tibia with/without IV contrast.

**Figure 3:**
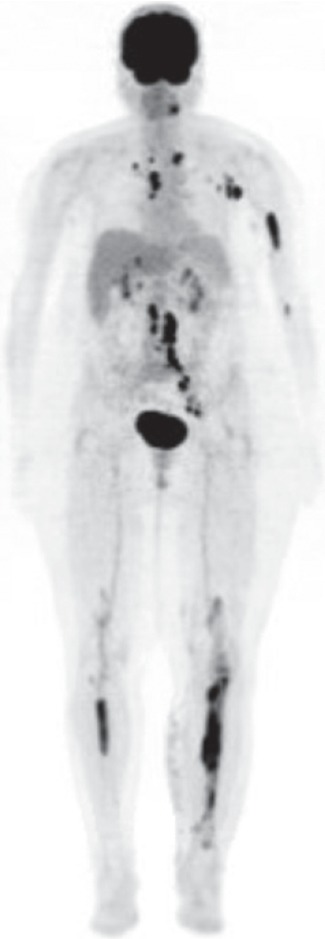
Whole body PET/CT scan.

A plain radiograph of the left leg (Figure 1) demonstrated increased lucency in the medullary canal extending from the proximal diaphysis of the mid-shaft with normal cortical appearance and no periosteal reaction or evidence of fractures. Magnetic resonance imaging (MRI) of the left leg revealed findings consistent with diffuse intramedullary metastasis involving the left tibia, visualized distal left femur, and proximal/mid-right tibia (Figure 2). Computed tomography (CT) scans of the chest, abdomen, and pelvis did not show any evidence of metastatic disease but demonstrated a few scattered nonspecific small mediastinal lymph nodes measuring up to 11  mm, which were likely reactive. The patient subsequently underwent an open tibial bone biopsy with tumor debulking and resection, along with prophylactic intramedullary nail placement of the left lower extremity. Pathology results of the bone biopsy were consistent with aggressive large B-cell non-Hodgkin's lymphoma (NHL). Neoplastic cells were found to be positive for CD3, Pax-5, CD20, MUM1, and BLC-2, while negative for CD10 and BLC-6. Ki-67 expression was high at greater than 95%. Following this new diagnosis, she underwent a positron emission tomography with computed tomography (PET/CT) scan which reported extensive disease involvement in both tibias, left humerus, and likely left mandible, as well as extensive adenopathy which extended from the supraclavicular region to the distal left external iliac system, believed to be due to her lymphoma (Figure 3).

#### 1.1.2. Case 2

A 51-year-old female presented to the emergency department with gradually progressive right knee pain in the summer of 2014. Plain radiography of her right femur showed a mottled appearance of the cortex and intramedullary spaces which was concerning for malignancy. MRI of the right femur showed an abnormal intramedullary signal and enhancement involving the distal femur with associated abnormal periosteal soft tissue, suggestive of an underlying lymphoma.

She underwent an open biopsy of the right distal femur and had pathology results consistent with kappa-restricted B-cell non-Hodgkin's lymphoma with high-grade features and significant necrosis. Neoplastic cells were positive for CD19, CD 20, PAX 5, and CD 10 and negative for CD23, CD5, and CD103. The Ki-67 index was more than 80%. Bone marrow aspiration and biopsy showed mild hypercellularity with trilineage hematopoiesis, but there was no evidence of lymphoma infiltration. PET/CT imaging demonstrated intense metabolic activity in the distal part of the right femur measuring 13.7 × 9.4 × 8.6  cm and a maximum standardized uptake value (SUV) of 21.3. There was also noted uptake in the medial aspect of the right thigh as well as intense metabolic activity in the right breast. Further investigation with MRI revealed suspicious lesions of the right breast at 8 o' clock and 11 o' clock positions, measuring 1.2 and 1.9  cm, respectively. She underwent ultrasound-guided biopsy of these lesions and had pathology results consistent with high grade B-cell non-Hodgkin lymphoma (NHL). CD20, PAX 5, and B-cell markers were strongly positive. Ki-67 showed a very high nuclear proliferative index with positive staining in 80–90% of the cells. The staining pattern for BCL-6, CD10, and MUM1 suggested a nongerminal center subtype. Fluorescent in situ hybridization (FISH) showed evidence of cMYC mutation, but t(14;18) was not observed.

The patient completed 6 cycles of dose-adjusted chemotherapy with rituximab, etoposide phosphate, prednisone, vincristine sulfate, cyclophosphamide, and doxorubicin (i.e., R-EPOCH). Restaging CT scans had demonstrated complete remission. She is currently under active surveillance with no evidence of disease recurrence since completing her chemotherapy regimen more than 3 years ago.

#### 1.1.3. Case 3

A 45-year-old male presented with right knee pain that was gradual in onset and not associated with any previous trauma. He was evaluated by orthopedic surgery and found to have a large effusion of the right knee. The patient subsequently underwent an MRI of the involved extremity that showed a diffuse heterogeneous marrow signal in the visualized distal femoral diaphysis extending to the femoral condyles with associated periosteal inflammation. Also noted was a fairly large soft tissue mass extending along the lateral and posterior aspects of the distal femoral diametaphysis. There was heterogeneous enhancement of the marrow process and a soft tissue mass which raised concern for an underlying neoplasm. CT scan of the chest did not show evidence of lymphadenopathy but did reveal a large mass extending from the posterior mediastinum around the right paraspinal soft tissues. Bone scan showed intense abnormal activity in the right distal femur. He underwent an open excisional biopsy of the right femoral mass which revealed kappa-restricted large B-cell NHL with high-grade features. Ki-67 expression was estimated to be between 80% and 90%. Cells partially expressed CD20 with loss of CD19. Immunohistochemical stains showed an expanded population of cells that were positive for CD45, CD20, PAX 5, CD10, and MUM1. Bone marrow biopsy and aspirate showed normocellular bone marrow without evidence of lymphoma through morphology or flow cytometry testing. Biopsy of the paraspinal mass revealed lambda-restricted B-cell NHL. The cytologic features in the paraspinal mass (predominance of small cells) and light chain restriction (lambda monotypia) were different than those observed in the right thigh mass (kappa-restricted large B-cell lymphoma). After confirmation with flow cytometry, he was diagnosed with unrestricted B-cell NHL and started treatment with a dose-adjusted R-EPOCH regimen. He completed 6 cycles of chemotherapy with partial response. His treatment course was complicated by avascular necrosis of the right distal femur. Although there has been persistence of the right paraspinal mass, he has had no evidence of disease progression close to 2 years after completion of chemotherapy.

#### 1.1.4. Case 4

A 28-year-old female had developed progressive right hip pain which acutely worsened after sustaining a fall while exiting her car and prompted her to go to the emergency department. Plain radiographs showed an impacted subcapital right hip fracture with multiple ill-defined lytic lesions throughout the femoral neck and intertrochanteric region, thus raising concern for a pathologic hip fracture. MRI of the hip showed areas of marrow replacement with enhancement throughout the proximal right femur as well as marrow enhancement within the superior and inferior pubic ramus, right sacrum, right iliac bone, and pubic symphysis. A bone scan was done that showed numerous foci of increased radiotracer activity in the right femur, left tibia, sternum, skull, and lumbar spine suggestive of metastatic disease. CT scan of the chest, abdomen, and pelvis demonstrated osseous destructive lesions with pathologic fractures at L2 and L4, as well as ill-defined hypodensities within the spleen. There was absence of lymphadenopathy noted on the CT scan. She underwent a total right hip replacement as well as biopsy of the bone lesion. Pathology results were consistent with diffuse large B-cell lymphoma with aggressive features and a germinal center subtype histology. The neoplastic cells demonstrated a lymphoid phenotype and expressed CD20 and PAX-5, as well as BCL-6, but were negative for CD10 and MUM1. Ki-67 staining was noted in more than 95% of the viable specimen. Tumor cells were negative for BCL-2 and cMYC. Core biopsy of the bone marrow demonstrated 95% cellularity with extensive diffuse pattern proliferation of neoplastic lymphocytes, consistent with large B-cell lymphoma infiltration of the bone marrow.

## 2. Discussion

The exact definition of PBL has remained a controversial topic throughout medical literature. The general consensus based on old WHO classifications and recommendations provided by the abovementioned IELSG 14 study has been to classify patients with osseous lesions that have no evidence of regional lymph node or distal visceral involvement within the category of PBL. There are currently no clearly defined risk factors associated with the development of this malignancy. Although there have been identified correlations between PBL and other bone disorders (e.g., Paget's disease and hereditary exostoses), infectious processes (e.g., human immunodeficiency virus (HIV) and osteomyelitis), and autoimmune disorders (e.g., sarcoidosis), none of these associations are well established as risk factors for the development of this disease [4–7].

### 2.1. Clinical Features

The median age range for patient's diagnosed with PBL varies between 45 and 60 years old, with fewer cases reported in the pediatric population [8]. The disease shows a male preponderance with a male-to-female ratio ranging from 1.2 to 1.8 [9]. There is insufficient data to provide any racial or geographical distribution of the disease. Pain (82–92%) and swelling (34–45%) of the involved site are two of the most common clinical manifestations of this disease [10]. Other less common presentations include pathological fractures and systemic “B-type” symptoms such as fevers, weight loss, and night sweats. Any skeletal site can be involved by PBL, but there is a higher rate of axial involvement as compared to the appendicular skeleton. When there is appendicular skeletal involvement, the femurs have proven to be most commonly affected, consisting of approximately 20–38% of cases [10]. Spread to lymph nodes and bone marrow occurs in about 28% and 35% of cases, respectively. Spinal cord compression is the most dreaded complication associated with this malignancy, occurring in almost 16% of patients. Osteolysis and resultant hypercalcemia is another major complication observed in approximately 5–10% of patients upon their initial presentation. It is absolutely pertinent that these medical emergencies are not overlooked within this patient population and that they are identified in a timely manner in order to prevent further complications.

#### 2.1.1. Diagnosis

Laboratory results are usually unremarkable and do not aid in the diagnosis of PBL. Initial diagnostic workup includes radiographic imaging of the affected area. However, radiographic findings for cases of PBL can be difficult to distinguish from other primary bone tumors such as osteosarcoma, Ewing's sarcoma, and chondrosarcoma. Plain X-ray films are the initial diagnostic test of choice. PBL most commonly presents as osteolytic or osteoblastic lesions with disease involvement of the cortex and reactive periosteal changes [11]. CT scan can be used to further delineate these lesions and remains the primary modality for staging, restaging, and follow-up of PBL. CT can accurately define the tumor boundaries and detect evidence of extraosseous extension as well as cortical breakthrough [12]. MRI further assists in diagnosis by revealing the extent of disease in greater detail through demonstration of abnormal signal intensity areas on T1- and T2-weighted images with minimal contrast enhancement. When comparing this imaging modality to PET/CT, MRI has proven to be equally effective at monitoring tumor response to treatment [13]. However, functional assessment of bone lesions using fluorodeoxyglucose (FDG)-PET imaging continues to play an important role. Studies have shown that FDG-PET displays a higher specificity and sensitivity than conventional bone scintigraphy in identifying lymphomatous infiltration of skeletal tissue [14]. In addition, sensitivity, specificity, and accuracy rates for staging of extranodal lymphomas with PET-CT have been reported to be 97%, 100%, and 98%, respectively, as compared to rates of 87%, 85%, and 84% using conventional CT imaging [15]. Despite the minimal availability of data, PET-CT is recommended as a standard tool for the initial evaluation, staging, and response assessment of FDG-avid lymphomas by the recent Lugano Classification System [16].

#### 2.1.2. Microscopy

Clinical and radiological suspicion for PBL should be further assessed by pathology via bone biopsy. It is recommended to avoid excisional biopsies and minimize the amount of resected tissue in order to reduce the risk of pathological fractures in these cases. The majority of PBL cases are diagnosed as diffuse large B-cell lymphoma (DLBCL) and less commonly as follicular lymphoma, small lymphocytic lymphoma, and marginal zone lymphoma [9].

Upon morphological inspection, tumor cells are large in size and appear consistent with follicle center or centroblastic cell types [17]. Flow cytometry demonstrates immunoreactivity for B-cell markers including CD45, CD20, CD21, CD45, and CD79a, with variable immunoreactivity for CD75 and CD10 [18, 19]. T-cell markers are usually negative using this technique, except for occasional small CD3 positivity. Available data on primary bone T-cell lymphomas are currently lacking with most reported cases being anaplastic large-cell lymphoma (CD3 +; CD43+; CD30+), which are often associated with *t* (2; 5) (p23; q35) and ALK-1 expression [20].

BCL2 and MYC rearrangements play an important role in tumor mechanisms. A study by Li et al., which studied the gene expression signatures of PBL, reported 6 out of 8 cases with BCL2 rearrangement while an additional 5 out of 17 cases were found to have MYC rearrangements. A case of dual MYC and BCL-2 gene rearrangements has also been reported. Thus far, BCL-6 rearrangements have not been shown to occur in patients with PBL [21].

#### 2.1.3. Staging and Prognostic Factors

Staging of PBL is required in order to provide appropriate therapy and avoid excessive treatment with antineoplastic agents which would potentiate the risk of developing serious adverse effects. The most commonly used staging criteria for PBL has been proposed by the Lugano Classification System [16]. Stage IE represents disease confined to an extranodal site, such as a solitary bone lesion. If there is evidence of regional lymph node involvement with a single bone lesion, the disease is then classified as stage IIE. Multifocal disease that is strictly limited to the skeletal system is classified as stage IV [22].

The prognosis of patients with primary bone DLBCL is directly correlated to the stage of disease. 5-year overall survival (OS) varies from 82% for patients with stage IE disease to 38% for cases of disseminated DLBCL with skeletal involvement. Conventional prognostic factors, such as the International Prognostic Index (IPI), have proven to be ineffective in predicting the prognosis for PBL since staging and the number of extranodal sites have no variability in PBL [23]. Importantly, however, the IELSG14 study revealed that patient age, functional performance status, and serum lactate dehydrogenase (LDH) levels are independently associated with OS in each subgroup of PBL. Furthermore, the germinal center (GC) phenotype with certain molecular features (i.e., CD10 expression, BCL-6 mutations, translocations involving 3q27) are associated with favorable outcomes in primary bone DLBCL [24]. On the other hand, non-GC signatures and related features (i.e., immunoblastic variant, MUM1 expression) are unfavorable predictors of survival in DLBCL of the bone [25]. Additionally, there is a worse prognosis associated with primary bone T-cell lymphomas as compared to primary bone B-cell lymphomas, especially in cases of CD56-positive anaplastic large-cell lymphomas in particular [26].

#### 2.1.4. Treatment

Given the rarity of this disease process, randomized-controlled clinical trials addressing treatment alternatives for PBL are not readily available. Therefore, current treatment recommendations are largely based upon data collected from retrospective studies. In the past, PBL was treated with radiation therapy alone which achieved appropriate localized disease control, but was associated with high relapse rates. Due to the elevated rate of disease recurrence, chemotherapy was introduced for the treatment of this malignancy [27]. Currently, there exist multiple treatment modalities for PBL including chemotherapy, localized radiation therapy, and surgical intervention. A retrospective multicenter Rare Cancer Network study involving 116 patients with stage IE (80% of cases) or IIE PBL demonstrated multiple factors associated with improved 5-year survival. These include an IPI score of less than 2, radiation dose greater than 40  Gy, and the administration of at least 6 cycles of chemotherapy [28]. The role of surgery is often limited to surgical biopsy with or without debulking and plays little role in cases of advanced disease. In some cases, patients with involvement of the weight-bearing bones may require internal stabilization or bracing until treatment and subsequent bone healing occur.

Most of PBLs have characteristics consistent with DLBCL; hence, chemotherapeutic regimens usually consist of anthracycline-based combination therapies such as cyclophosphamide, doxorubicin, vincristine, and prednisone (CHOP) with or without rituximab (R-CHOP). Of note, in a study involving 131 PBL patients treated with either CHOP or R-CHOP, Ramadan et al. demonstrated that R-CHOP was superior to CHOP with 3-year progression-free survival (PFS) rates of 88% as compared to 52%, respectively [29]. Treatment with radiation therapy dates to an era without the addition of rituximab to chemotherapy; thus, its use remains a topic of much debate. Prior to the introduction of rituximab, combination chemotherapy and radiotherapy was the treatment regimen of choice. In patients with unifocal disease, consolidative involved-field radiotherapy provides an adequate response. A dose of 30–36  Gy has been found to be effective, with higher doses (e.g., 40  Gy) offered to patients with an indeterminate response to radiation. However, attention should be drawn to the specific areas of disease involvement before pursuing treatment with radiotherapy. Radiation applied to skeletal structures involved in a significant amount of bone marrow production, such as the pelvis, should be cautiously considered in order to avoid complications with hematopoiesis.

Even with the limited data available, chemotherapy has proven to be more effective than radiation therapy for the treatment of PBLs with 10-year overall survival rates of 56% as compared to 25%, respectively. Yet, several studies have demonstrated improved overall survival when using combined chemoradiation rather than chemotherapy or radiation alone [30]. For example, a prospective study conducted by Christie et al. involving cases of PBL treated with three cycles of CHOP followed by 45  Gy of involved field radiation listed 5-year local control rates of 72% and overall survival rates of 90% [31]. Therefore, it is currently recommended that patients with diagnosed PBL receive treatment with combined chemoradiation in order to best achieve remission. Nevertheless, with the increasing development of novel systemic therapies, further research is warranted for the identification of treatment regimens that will improve progression-free and overall survival rates for patients suffering from PBL.
